# Metformin and Bone Metabolism in Endogenous Glucocorticoid Excess: An Exploratory Study

**DOI:** 10.3389/fendo.2021.765067

**Published:** 2021-10-27

**Authors:** Frederick Vogel, Leah Braun, German Rubinstein, Stephanie Zopp, Andrea Oßwald, Katharina Schilbach, Ralf Schmidmaier, Martin Bidlingmaier, Martin Reincke

**Affiliations:** Medizinische Klinik und Poliklinik IV, LMU Klinikum, Ludwig-Maximilians-Universität München, Munich, Germany

**Keywords:** metformin, hypercortisolism, glucocorticoids, bone density, osteoporosis, cortisol

## Abstract

**Context:**

Glucocorticoid excess exhibits multiple detrimental effects by its catabolic properties. Metformin was recently suggested to protect from adverse metabolic side-effects of glucocorticoid treatment. Whether metformin is beneficial in patients with endogenous glucocorticoid excess has not been clarified.

**Objective:**

To evaluate the phenotype in patients with endogenous Cushing’s syndrome (CS) treated with metformin at the time of diagnosis.

**Patients and Methods:**

As part of the German Cushing’s Registry we selected from our prospective cohort of 96 patients all 10 patients who had been on pre-existing metformin treatment at time of diagnosis (CS-MET). These 10 patients were matched for age, sex and BMI with 16 patients without metformin treatment (CS-NOMET). All patients had florid CS at time of diagnosis. We analyzed body composition, metabolic parameters, bone mineral density and bone remodeling markers, muscle function and quality of life.

**Results:**

As expected, diabetes was more prevalent in the CS-MET group, and HbA1c was higher. In terms of comorbidities and the degree of hypercortisolism, the two groups were comparable. We did not observe differences in terms of muscle function or body composition. In contrast, bone mineral density in metformin-treated patients was superior to the CS-NOMET group at time of diagnosis (median T-Score -0.8 *versus* -1.4, *p* = 0.030). CS-MET patients showed decreased β-CTX levels at baseline (*p* = 0.041), suggesting reduced bone resorption under metformin treatment during glucocorticoid excess.

**Conclusion:**

This retrospective cohort study supports potential protective effects of metformin in patients with endogenous glucocorticoid excess, in particular on bone metabolism.

## Introduction

Metabolic side-effects of glucocorticoids (GC) are common and challenging in both endogenous and exogenous GC excess. Patients with endogenous Cushing’s syndrome (CS) typically show comorbidities like arterial hypertension, visceral obesity, dyslipidemia, muscle dysfunction, osteoporosis and impaired glucose metabolism (1). CS is associated with poor quality of life, morbidity and increased mortality, even after successful surgery leading to biochemical remission (2–4). In a recently published study, Pernicova et al. (5) reported that metformin administration improved metabolic profiles of glucocorticoid-treated patients with inflammatory diseases in a randomized, double-blind, placebo-controlled, phase 2 clinical trial. Metformin treatment was associated with favorable effects on lipid profile, liver function, appetite, intima-media thickness and bone mineral density as well as bone turnover (5). However, whether metformin has beneficial effects in patients with endogenous GC excess is largely unknown. In patients with type 2 diabetes mellitus, metformin is the most widely used oral antihyperglycemic agent. Recently, new potential therapeutic applications in non-diabetic patients have been described, such as cardioprotection (6, 7), major depressive disorder (8) and cancer (9–11). The mechanisms of action are still not fully understood. The aim of this retrospective cohort study was to analyze the metabolic profile and bone turnover of patients with and without pre-existing metformin treatment at the time of endogenous GC excess. We hypothesized that standard metformin use is beneficial for bone metabolism in patients with florid CS.

## Patients and Methods

### Patients

**Figure 1:**
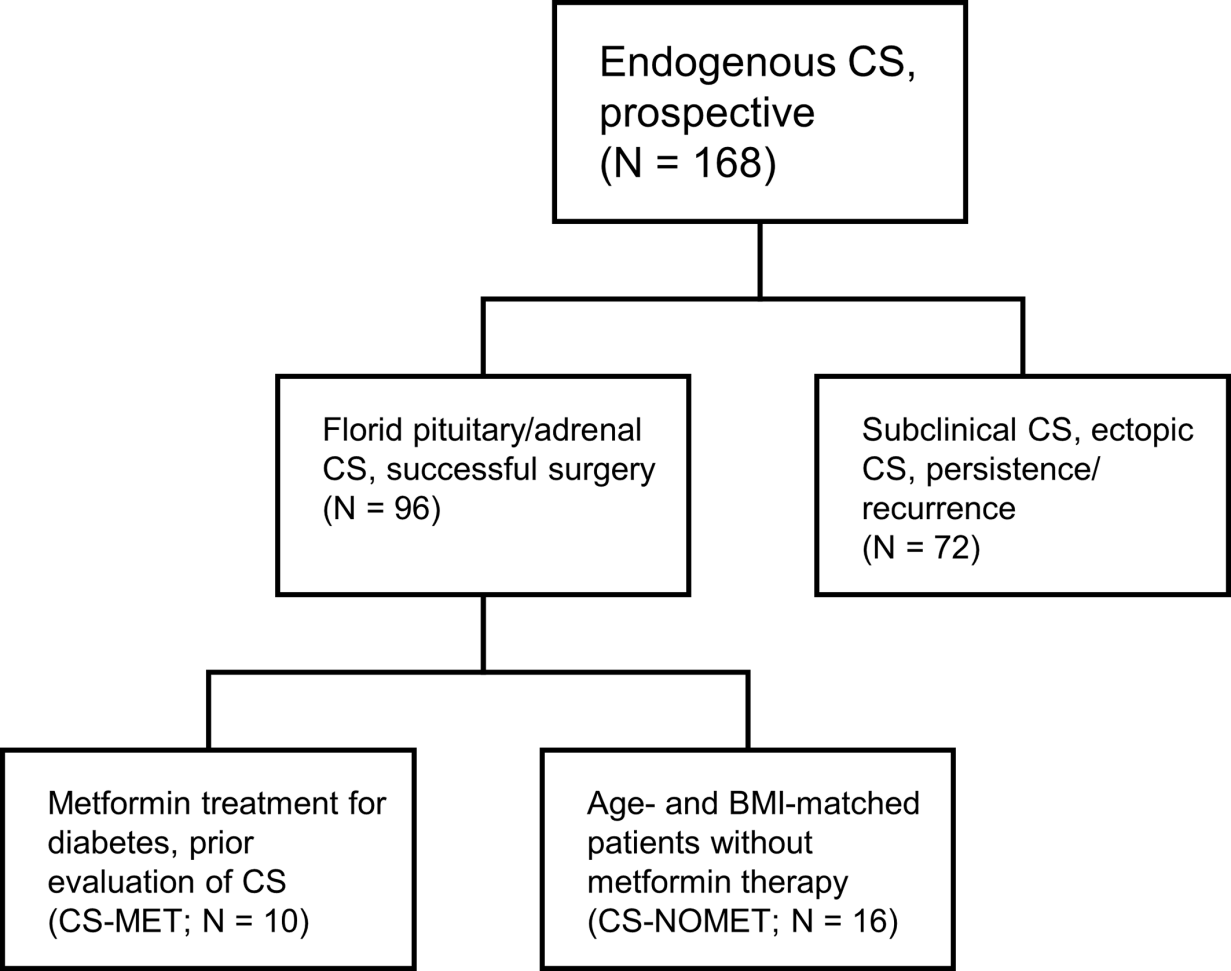
Patient selection. CS, Cushing’s syndrome; BMI, body mass index.

This cohort study was performed as part of the German Cushing’s Registry. General characteristics of the registry have been described in detail previously (12–14). We screened the prospective registry cohort consisting of 96 patients with endogenous CS for metformin intake at the time of diagnosis. Inclusion criteria for the current study were florid pituitary or adrenal CS, successful surgery leading to biochemical remission; exclusion criteria were subclinical hypercortisolism, ectopic CS, persistent/recurrent CS, adrenostatic or radiation therapy. We identified 10 patients who were taking metformin at the time of diagnosis of CS (CS-MET group). The mean metformin dose at the time of diagnosis was 1670 ± 472 mg per day. Metformin was initiated as routine therapy for diabetes at least 3 months prior evaluation of CS. For comparison, we selected 16 patients without metformin therapy at the time of diagnosis of CS and afterwards (CS-NOMET group). Matching was done according to age, body mass index (BMI), sex and subtype of CS. Patient selection is shown in **Figure 1**. All 26 patients had biochemically confirmed and clinically florid CS, diagnosed between 2012 and 2019 at Ludwig-Maximilian-University Munich. Diagnosis and subtype differentiation of CS were done as reported earlier according to the current guidelines and recommendations (12, 15). One year after successful surgery patients were re-evaluated clinically and biochemically in a standardized fashion. In CS-MET group, 9 out of 10 patients continued metformin therapy until one-year follow-up. For the comparison of bone remodeling markers, a previously described registry control group of patients in whom CS was excluded (NO-CS group, n=95) was used (14). The German Cushing’s Registry (NeoExNet, No. 152-10) was approved by the LMU ethics committee, and all patients gave written informed consent.

### Laboratory Analysis

In all patients, blood samples were taken in a fasting state at the time of diagnosis and one year after successful surgery in line with the follow-up visit. The analyses of standard laboratory values were performed in the central laboratory of the LMU Klinikum Munich using standard methods. The bone formation marker intact procollagen I-N-propeptide (PINP) and the bone resorption marker β-CTX (CrossLaps) were measured at the Endocrine Laboratory of Department of Medicine IV. The samples were centrifuged within 20 minutes, stored at -80° and then measured on the iSYS automated analyzer (IDS-iSYS, Boldon, UK) by validated assays (16–18).

### Bone Density and Muscle Strength Measurements

Bone mineral density (BMD) was measured at the lumbar spine and the femur (GE Lunar Prodigy Advance). Minimal T-Score was determined from both measurements using a gender-specific reference cohort as previously reported (14). BMD data at the time of diagnosis was available in 8 of 10 patients of CS-MET group and 13 of 16 patients of CS-NOMET group. For the assessment of muscle function, hand grip strength was measured three times on both hands per visit in a sitting position. The measurements were performed in a standardized manner with the JAMAR hydraulic hand dynamometer (Patterson Medical, Nottinghamshire, UK), as previously described (19). To adjust for age and gender (normalized grip strength) grip strength was standardized to the manufacturer’s information on normative grip strength data (20).

### Biometrics and Bio-Impedance Measurements

Bio-impedance and anthropometric measurements like BMI, waist-to-arm-ratio and waist-to-hip-ratio were performed by the same investigator in a standardized manner. Body cell mass and body fat percentage was estimated by using a bio-impedance measuring device at 50 kHz with 400 µA by Data Input (Poecking, Germany), according to the manufacturer’s information. Two pairs of current-introducing and voltage-sensing electrodes were attached to the dorsum of hand and foot. All impedance measurements were taken after fasting, the arms relaxed at the sides without touching the body.

### Quality of Life

To analyze quality of life in patients with CS, we used the disease-specific questionnaire Cushing’s quality of life (CushingQoL) (21). In addition, for quantification of depressive symptoms, Beck’s Depression Inventory was evaluated at the time of diagnosis and one year in remission of CS.

### Statistical Evaluation

Statistical analysis was performed using SPSS (version 26). Patient characteristics are shown as median and 25^th^ and 75^th^ percentile in brackets. For comparison between baseline and follow-up Wilcoxon signed rank test was used. Differences between the groups were analyzed using Mann-Whitney-U-Test. *P*-values of ≤ 0.05 were considered to indicate statistical significance.

## Results

### Patient Characteristics

Clinical and biochemical characteristics of the two patient groups are shown in **Table 1**, and anthropometric data is shown in **Table 2**. Cortisol concentrations in urinary free cortisol (UFC), late night salivary cortisol (LNSC) and 1 mg dexamethasone suppression test (DST) did not differ between the two groups at baseline and during follow-up (**Table 1**).

**Table 1:** Baseline and 1-year follow-up characteristics of patients with CS ± metformin.

Patient Characteristics	CS with metformin (CS-MET, n = 10)			CS without metformin (CS-NOMET, n = 16)			
	Baseline	After surgery	*P vs.* BL	Baseline	After surgery	*P vs.* BL	P*
Sex, female/male, n (%)	8 (80%)/2 (20%)	—	—	14 (87%)/2 (13%)	—	—	—
Diagnosis, pituitary/adrenal, n (%)	6 (60%)/4 (40%)	—	—	9 (56%)/7 (44%)	—	—	—
Age, years	59 [52; 64]	—	—	52 [39; 58]	—	—	0.165
Postmenopausal, n (% of female)	7 (88%)	—	—	10 (71%)	—	—	—
Vitamin D supplementation, n (%)	2 (20%)	—	—	8 (50%)	—	—	—
Metformin dose, mg per day	2000 [1000; 2000]	2000 [963; 2000]	0.458	—	—	—	—
Vitamin D, ng/mL	22 [16; 30]	25 [14; 39]	0.507	25 [18; 34]	32 [27; 42]	**0.017**	0.336
HbA1c, %	7.3 [6.9; 9.3]	6.3 [5.7; 6.7]	**0.005**	6.2 [5.7; 6.6]	5.5 [5.2; 5.9]	**0.002**	**0.001**
UFC, µg/24h	244 [163; 486]	20 [7; 36]	**0.018**	313 [138; 773]	24 [10; 38]	**0.001**	0.660
DST 1 mg	14.7 [7.3; 24.6]	—	—	11.3 [6.5; 18.1]	—	—	0.484
LNSC, ng/mL	6.0 [3.0; 10.0]	0.7 [0.5; 1.1]	**0.018**	4.3 [2.7; 7.2]	1.0 [0.6; 1.2]	**0.001**	0.660
ACTH in pituitary CS, pg/mL	60 [31; 93]	18 [11; 40]	0.173	69 [62; 118]	11 [8; 17]	**0.012**	0.328
ACTH in adrenal CS, pg/mL	4 [2; 5]	20 [13; 31]	0.068	4 [2; 5]	27 [9; 33]	**0.028**	0.927

Data are given as median and 25th and 75th percentile in brackets. Bold p-values indicates statistical significance. *CS-MET vs CS-NOMET at baseline. Comparisons between baseline and follow-up were performed by a Wilcoxon signed rank test, comparisons between groups at baseline with Mann-Whitney-U-Test.

CS, Cushing’s syndrome; BL, baseline; HbA1c, hemoglobin A1c; UFC, urinary free cortisol; LNSC, late night salivary cortisol; DST, dexamethasone suppression test.

**Table 2:** Anthropometric and musculoskeletal characteristics at baseline and 1-year follow-up of patients with CS ± metformin.

Patient Characteristics	CS with metformin (CS-MET; n = 10)			CS without metformin (CS-NOMET; n = 16)			
	Baseline	After surgery	*P vs.* BL	Baseline	After surgery	*P vs.* BL	P*
BMI, kg/m^2^	37 [29; 43]	33 [29; 36]	**0.013**	33 [31; 43]	31 [27; 35]	**0.002**	0.586
Waist-to-hip-ratio	1.1 [1.0; 1.2]	1.0 [0.9; 1.1]	0.213	1.0 [0.9; 1.1]	0.9 [0.8; 1.0]	**0.026**	**0.041**
Waist-to-arm-ratio	3.8 [3.2; 4.7]	3.6 [3.3; 3.9]	**0.037**	3.5 [3.2; 3.7]	3.2 [2.8; 3.3]	**0.039**	0.077
BMD lumbar spine (T-Score)	0.2 [-0.5; 2.8]	—	—	-1.1 [-2.0; 0.2]	—	—	**0.037**
BMD femur (T-Score)	-0.8 [-0.9; -0.2]	—	—	-1.3 [-1.8; -0.2]	—	—	0.238
Body fat, %	37 [28; 47]	31 [23; 42]	**0.050**	40 [34; 49]	36 [30; 37]	0.075	0.431
Muscle mass, kg	31 [23; 39]	30 [24; 32]	0.225	29 [22; 32]	29 [26; 32]	0.273	0.639
Grip strength, % of normal controls	95 [77; 113]	67 [54; 93]	0.169	84 [68; 99]	79 [50; 97]	0.079	0.363

Data are given as median and 25th and 75th percentile in brackets. Bold p-values indicates statistical significance. *CS-MET vs CS-NOMET at baseline. Comparisons between baseline and follow-up were performed by a Wilcoxon signed rank test, comparisons between groups at baseline with Mann-Whitney-U-Test.

CS, Cushing’s syndrome; BL, baseline; BMI, body mass index; BMD, bone mineral density.

### Diabetes, BMI, and Body Composition

At baseline, all 10 patients of CS-MET group had confirmed diabetes, compared to 4 of 16 in the CS-NOMET group and, thus, had higher HbA1c levels (*p* = 0.001, **Table 1**). No relevant difference between the two groups was present at baseline in terms of BMI, body fat percentage and estimated muscle mass by bio-impedance measurements (**Table 2**). One year after remission, BMI and HbA1c had decreased in both groups. Compared to CS-NOMET group, metformin-treated patients showed a reduction in body fat percentage following remission that was borderline significant (*p* = 0.050, **Table 2**).

### Bone Mineral Density and Muscle Function

**Figure 2:**
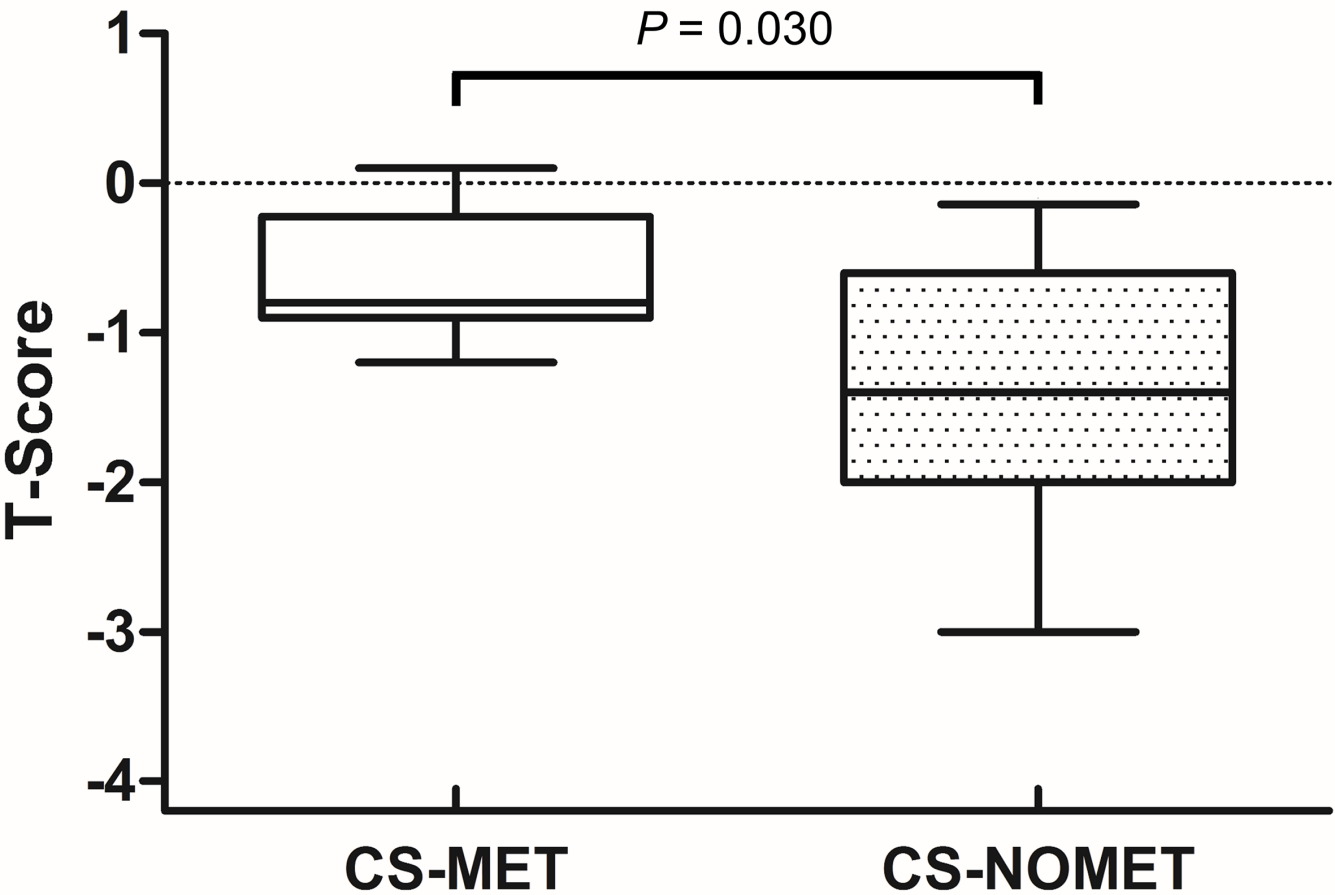
Bone mineral density (T-Scores) in patients with florid Cushing’s syndrome (CS) and pre-existing metformin therapy (CS-MET) or without pre-existing metformin (CS-NOMET). Boxplot = median and ranges of T-Scores. CS-MET: n = 8; CS-NOMET: n = 13. Comparison between groups by Mann-Whitney-U-Test; *p ≤* 0.05 was considered statistically significant.

**Figure 3:**
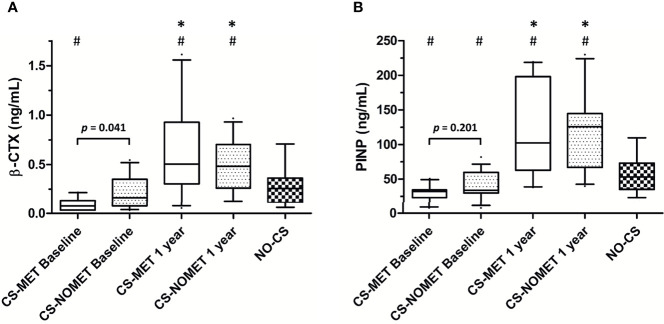
Bone turnover markers during glucocorticoid excess and one year after surgically induced remission in patients with endogenous Cushing’s syndrome (CS). Pre-existing metformin treatment (CS-MET): n = 10; no pre-existing metformin treatment (CS-NOMET): n = 16; patients with excluded CS (NO-CS): n = 95. **(A)** β-CTX = β-Crosslaps; **(B)** PINP = procollagen I-N-propeptide. Box and whiskers (10-90 percentile). Comparison between groups was performed by a Mann-Whitney-U-Test, between time points by a Wilcoxon signed rank test; p ≤ 0.05 was considered statistically significant. *p ≤ 0.05 *versus* baseline; ^#^ *p ≤* 0.05 *versus* ‘NO-CS’.

At the time of diagnosis, vitamin D serum concentrations were similar between groups, and no patient had bisphosphonate or denosumab treatment. BMD in metformin-treated patients was higher compared to patients without metformin (median T-Score -0.8 in CS-MET group versus -1.4 in CS-NOMET, *p* = 0.030, **Figure 2**), and the concentration of the bone resorption marker β-CTX at baseline was lower in the CS-MET group than in the CS-NOMET group (*p* = 0.041, **Figure 3A**). PINP, a bone formation marker, showed no difference between the two groups (*p* = 0.201, **Figure 3B**). One year after successful surgery both bone markers strikingly increased, with no difference between CS-MET and CS-NOMET group. Differences in muscle function measured by grip strength did not reach statistical significance. However, patients with metformin had a trend to less muscular impairments during GC excess (**Table 2**).

## Discussion

This exploratory cohort study analyzed the metabolic effect of metformin intake at time of diagnosis in patients with endogenous GC excess. Our results suggest that metformin has a beneficial effect on bone metabolism during endogenous hypercortisolism.

In a recently published study we showed that during florid CS bone metabolism is characterized by decreased bone formation and increased bone resorption, followed by a strong activation of bone turnover after successful treatment inducing biochemical remission of CS (14). In the present study, metformin-treated patients had better BMD and lower serum β-CTX concentrations, indicating decreased bone resorption during hypercortisolism compared to patients with florid CS and no metformin therapy. Likewise, Pernicova and colleagues observed in their randomized study decreased bone resorption markers and increased BMD in metformin-treated patients compared to placebo-treated patients, all receiving exogenous glucocorticoids (5). The results of our study are in line with a beneficial effect on bone metabolism during GC excess and can be interpreted that also patients with endogenous CS may benefit from metformin administration. Decreased concentrations of the bone resorption marker β-CTX under metformin treatment were already reported in patients with type 2 diabetes (22). Furthermore, metformin was shown to have osteogenic effects *in vitro* and *in vivo via* an increase in the expression of Runx2 and in the phosphorylation/activation of AMP-activated-protein-kinase (AMPK) (23, 24). Metformin was recently reported to improve the osteogenic differentiation potential of bone marrow-derived mesenchymal stem cells from patients with type 2 diabetes (25). However, metformin intake was as well associated with decreased bone formation markers, such as PINP, contrary to animal studies (22). Our study showed slightly lower PINP concentrations in the metformin-treated group, without reaching statistical significance. Whether metformin treatment is associated with a reduced risk of fractures remains controversial (26). Furthermore, metformin was shown to improve liver metabolism during GC excess (5), which in turn could have positive effects on bone health (27, 28).

In a recently conducted study on the long-term outcome of CS associated myopathy we identified age, HbA1c and waist-to-hip-ratio as predictors of myopathy outcome in patients with CS in remission (19). Despite a more pronounced hyperglycemic state (**Supplementary Table 1**), patients with metformin in our study showed a trend to less muscular impairment in grip strength during hypercortisolism (**Table 2**). Whether metformin administration generally blunts or improves exercise training-induced effects on skeletal muscle is controversial (29–31). Quality of life and depressive symptomatology revealed no significant differences between the two study groups (**Supplementary Table 1**). However, metformin showed antidepressant effects in larger cohorts and animal models (8, 32), and was even shown to correct abnormal circadian rhythm on a cellular level *via* an activation of AMPK (33). Mechanistically, the action of metformin is not yet fully understood. AMPK pathway is suggested to be a key mediator of glucocorticoid-metformin interaction (5, 34–36). In addition, metformin could show positive and protective effects during endogenous GC excess *via* an increase of serum insulin-like growth factor-I (37) and fibroblast growth factor 21 (38–40), and/or *via* a suppression of neuroendocrine tumor growth (9, 41, 42). However, the underlying mechanism behind a potential protection against glucocorticoid-associated adverse side-effects remains largely unknown.

So far, a potent agent reducing adverse GC side-effects is lacking. Further studies are required to investigate the effect during hypercortisolism and on long-term outcome and persistent symptoms such as myopathy, cardiovascular risk and cognitive disorders. Clearly, there is a need for a prospective randomized controlled trial on the effect of metformin in patients with endogenous CS.

### Strength and Limitations of the Study

Because of the retrospective selection of patients with pre-existing metformin treatment at the time of diagnosis, groups differ regarding hyperglycemia. The interpretation of metabolic profiles should thus be done with caution. Another limitation is the small number of patients that follows from the rarity of the disease, and, due to the retrospective study design, the unknown duration of metformin therapy prior to diagnosis of CS. Although BMI was not significantly different between the two groups, increased truncal obesity in patients with metformin could positively affect BMD. On the other hand, however, diabetes is a known risk factor for osteoporosis *per se*, which further emphasizes the protective effect of metformin. Moreover, a strength of the study is the pre-existing metformin treatment, in the way that metformin intake already existed during the pathogenesis and development of endogenous GC excess.

## Conclusion

This study supports the concept that metformin has a protective effect on bone metabolism in patients with endogenous glucocorticoid excess.

## Data Availability Statement

The original contributions presented in the study are included in the article/**Supplementary Material**. Further inquiries can be directed to the corresponding author.

## Ethics Statement

The studies involving human participants were reviewed and approved by LMU ethics committee. The patients/participants provided their written informed consent to participate in this study.

## Author Contributions

FV served as the principal investigator in this work and was responsible for the study conception and design, the analysis and interpretation of the data, and the drafting of the manuscript. GR, SZ, and AO contributed to the collection and analysis of the data. KS, RS, and MB substantially contributed to the interpretation of the data and the drafting of the manuscript. MR contributed to the conceptual design of the study, the collection, analysis and interpretation of data, and the drafting of the paper. All authors contributed to the article and approved the submitted version.

## Funding

This work is part of the German Cushing’s Registry CUSTODES and has been supported by a grant from the Else Kröner-Fresenius Stiftung to MR (2012_A103 and 2015_A228). MR and AO are supported by the Deutsche Forschungsgemeinschaft (DFG, German Research Foundation, project number: 314061271-TRR 205). LB is supported by the Clinician Scientist Program RISE (Rare Important Syndromes in Endocrinology), supported by the Else-Kröner-Fresenius Stiftung and Eva Luise und Horst Köhler Stiftung. FV is supported by the Deutsche Forschungsgemeinschaft (DFG, German Research Foundation, project number: 413635475) and the Munich Clinician Scientist Program (MCSP) of the LMU München.

## Conflict of Interest

The authors declare that the research was conducted in the absence of any commercial or financial relationships that could be construed as a potential conflict of interest.

## Publisher’s Note

All claims expressed in this article are solely those of the authors and do not necessarily represent those of their affiliated organizations, or those of the publisher, the editors and the reviewers. Any product that may be evaluated in this article, or claim that may be made by its manufacturer, is not guaranteed or endorsed by the publisher.
