# Supplementary material for: Metformin and Bone Metabolism in Endogenous Glucocorticoid Excess: An Exploratory Study
Source: Front Endocrinol (Lausanne). 2021 Oct 27;12:765067. doi: 10.3389/fendo.2021.765067 (PMC8578886; doi:10.3389/fendo.2021.765067)
Supplement: Supplementary file 1 [file Table_1.docx]

| **Supplement Table 1**. Baseline and 1-year follow-up characteristics of patients with CS ± metformin. | | | | | | | |
| --- | --- | --- | --- | --- | --- | --- | --- |
|  | CS with metformin  (CS-MET; n=10) | | | CS without metformin  (CS-NOMET; n=16) | | |  |
| **Patient Characteristics** | **Baseline** | **After surgery** | ***P* vs BL** | **Baseline** | **After surgery** | ***P* vs BL** | ***P* *** |
| FPG, mg/dL | 140 [107; 197] | 124 [84; 158] | 0.139 | 96 [89; 106] | 90 [82; 99] | 0.219 | **0.004** |
| Insulin, µIU/mL | 20 [11; 36] | 19 [7; 46] | 0.374 | 14 [9; 18] | 9 [5; 16] | **0.045** | 0.379 |
| HOMA-Index | 8.2 [2.9; 11.9] | 6.3 [1.3; 15.1] | 0.906 | 3.5 [2.6; 5.1] | 2.3 [1.2; 4.4] | 0.100 | 0.084 |
| TSH, µU/mL | 1.23 [0.83; 1.63] | 2.00 [0.94; 2.87] | 0.285 | 1.06 [0.78; 1.61] | 1.5 [0.6; 2.4] | 0.255 | 0.660 |
| WBC, G/L | 9.4 [7.4; 11.7] | 9.3 [5.8; 9.6] | **0.017** | 8.7 [6.8; 10.3] | 7.0 [5.4; 7.9] | **0.001** | 0.182 |
| CRP, mg/dL | 0.12 [0.05; 0.44] | 0.34 [0.25; 0.68] | 0.066 | 0.35 [0.10; 0.72] | 0.47 [0.14; 0.78] | 0.308 | 0.103 |
| Cholesterol, mg/dL | 202 [147; 236] | 211 [156; 235] | 0.683 | 213 [194; 241] | 215 [187; 235] | 0.073 | 0.262 |
| Triglycerides, mg/dL | 210 [140; 362] | 259 [183; 339] | 0.285 | 147 [87; 173] | 142 [108; 163] | 0.433 | **0.041** |
| HDL, mg/dL | 46 [38; 62] | 42 [36; 59] | 0.262 | 65 [54; 70] | 55 [48; 64] | 0.184 | **0.006** |
| LDL, mg/dL | 100 [73; 157] | 129 [64; 153] | 0.959 | 123 [106; 158] | 118 [106; 141] | 0.084 | 0.182 |
| Gamma-GT, U/L | 63 [25; 100] | 40 [21; 50] | 0.128 | 35 [21; 47] | 17 [14; 30] | **0.001** | 0.267 |
| AST, U/L | 21 [19; 30] | 21 [17; 27] | 0.207 | 20 [17; 26] | 20 [18; 25] | 0.378 | 0.636 |
| ALT, U/L | 33 [24; 48] | 23 [15; 31] | **0.012** | 30 [25; 39] | 19 [13; 27] | **0.006** | 0.776 |
| AST/ALT ratio | 0.84 [0.54; 0.90] | 0.99 [0.80; 1.22] | **0.017** | 0.74 [0.61; 0.83] | 1.06 [0.90; 1.46] | **0.001** | 0.357 |
| Antidepressants, n (%) | 4 (40%) | — | — | 4 (25%) | — | — | — |
| Thyroid hormone replacement, n (%) | 4 (40%) | — | — | 8 (50%) | — | — | — |
| Insulin therapy, n (%) | 2 (20%) | — | — | 1 (6%) | — | — | — |
| PPI therapy, n (%) | 2 (20%) | — | — | 3 (19%) | — | — | — |
| Hypertension | 10/10  (100 %) | — | — | 15/16  (94 %) | — | — | — |
| SBP, mmHg | 130 [123; 140] | 132 [109; 142] | 0.594 | 144 [133; 159] | 122 [117; 137] | **0.003** | **0.036** |
| DBP, mmHg | 81 [75; 89] | 77 [70; 91] | 0.386 | 90 [85; 101] | 79 [76; 90] | **0.005** | **0.031** |
| Antihypertensive agents | 3 [3; 3] | 2 [1; 3] | **0.019** | 2 [1; 3] | 1 [0; 2] | **0.004** | **0.041** |
| Smokers | 3/10 (30 %) | — | — | 4/16 (25 %) | — | — | — |
| CushQoL (range 0-100) | 45 [29; 53] | 59 [45; 74] | 0.078 | 35 [24; 50] | 46 [32; 72] | 0.071 | 0.639 |
| BDI (range 0-63) | 9 [6; 19] | 10 [6; 21] | 0.102 | 19 [9; 28] | 11 [7; 21] | 0.126 | 0.208 |

Data are given as median and 25th and 75th percentile in brackets. Bold *p*-values indicates statistical significance. * CS-MET vs CS-NOMET at baseline. Comparisons between baseline and follow-up were performed by a Wilcoxon signed rank test, comparisons between groups at baseline with Mann-Whitney-U-Test.

Abbreviations: CS, Cushing’s syndrome; BL, baseline; FPG, fasting plasma glucose; HOMA, Homeostasis Model Assessment; WBC, white blood cells; CRP, c-reactive protein; AST = aspartate aminotransferase; ALT = alanine aminotransferase; Gamma-GT = Gamma-glutamyl transferase; PPI, proton pump inhibitor; SBP, systolic blood pressure; DBP, diastolic blood pressure; CushQoL, Cushing’s quality of life; BDI, Beck Depression Inventory.
